# Prevalence and seasonal aspects of parasitism by *Oestrus ovis* (Diptera: Oestridae) in sheep from Mato Grosso State, Brazil

**DOI:** 10.1590/S1984-29612024020

**Published:** 2024-04-22

**Authors:** Victória Luiza de Barros Silva, Dirceu Guilherme de Souza Ramos, Richard de Campos Pacheco, Diego Montagner Schenkel, Nilton Pereira Dias, Artur Kanadani Campos, Fernando Henrique Furlan

**Affiliations:** 1 Laboratório de Parasitologia Veterinária e Doenças Parasitárias dos Animais Domésticos e Silvestres, Hospital Veterinário - HOVET, Faculdade de Medicina Veterinária - FAVET, Universidade Federal de Mato Grosso - UFMT, Cuiabá, MT, Brasil; 2 Laboratório de Parasitologia e Análises Clínicas Veterinária, Unidade Acadêmica de Ciências Agrárias, Universidade Federal de Jataí - UFJ, Jataí, GO, Brasil; 3 Hospital Veterinário - HOVET, Instituto de Ciências da Saúde - ICS, Universidade Federal de Mato Grosso - UFMT, Sinop MT, Brasil; 4 Laboratório de Patologia Veterinária, Hospital Veterinário - HOVET, Faculdade de Medicina Veterinária - FAVET, Universidade Federal de Mato Grosso - UFMT, Cuiabá, MT, Brasil

**Keywords:** Sheep, Mato Grosso, myiasis, oestrosis, Ovino, Mato Grosso, miíase, oestrose

## Abstract

*Oestrus ovis* larvae are obligate parasites of vertebrates and cause cavitary myiasis (oestrosis) in sheep and goats. It is also reported as a zoonosis causing ophthalmomyiasis and nasopharyngeal myiasis. Despite being relatively common in Brazil, epidemiological studies on *O. ovis* are scarce. Considering that the infestation is influenced by the climate and biomes of the studied region, we aimed to investigate the seasonal prevalence of *O. ovis* among slaughtered sheep in the northern region of the state of Mato Grosso, Brazil. The heads of sheep (n=697) slaughtered at a slaughterhouse in the municipality of Terra Nova do Norte (November 2011 to November 2013) were collected to count, catalog, and identify the larvae found in the upper respiratory tract. Overall, 45.77% (319/697) of the animals were infested with 2,412 recovered larvae, 96.89% (2,337/2,412) of which were identified at the species level as *O. ovis*. Seasonal variations in prevalence ranged from 41% (spring) to 56% (summer); however, no correlation was observed between prevalence and season, mean humidity, or temperature. In conclusion, parasitism by *O. ovis* in sheep in the studied area, occurs year-round, considering the occurrence of larvae (L1, L2, and L3) throughout the year, probably because of the area's environmental conditions.

## Resumo

As larvas de *Oestrus ovis* são parasitas obrigatórios de vertebrados e causam miíase cavitária (estrose) em ovinos e caprinos. Também é relatada como uma zoonose que causa oftalmomíase e miíase nasofaríngea. Apesar de relativamente comum no Brasil, os estudos epidemiológicos sobre *O. ovis* são escassos. Considerando que a infestação é influenciada pelo clima e biomas da região estudada, objetivou-se investigar a prevalência sazonal de *O. ovis* entre ovinos abatidos na região norte do estado de Mato Grosso, Brasil. Foram coletadas cabeças de ovinos (n=697) abatidos em frigorífico do município de Terra Nova do Norte (novembro de 2011 a novembro de 2013) para contagem, catalogação e identificação das larvas encontradas no trato respiratório superior. No geral, 45,77% (319/697) dos animais estavam infestados com 2.412 larvas recuperadas, das quais 96,89% (2.337/2.412) foram identificadas em nível de espécie como *O. ovis*. As variações sazonais na prevalência variaram de 41% (primavera) a 56% (verão); no entanto, não foi observada correlação entre prevalência e estação do ano, umidade média ou temperatura. Conclui-se que o parasitismo por *O. ovis* em ovinos na área estudada ocorre durante todo o ano, considerando a ocorrência de larvas (L1, L2 e L3) ao longo do ano, provavelmente devido às condições ambientais da área.

## Introduction

Among the several forms of parasitism, ectoparasitism is one of the most important and includes the Order Diptera, which affects the livestock industry and production. *Oestrus ovis* (Linné, 1758) (Diptera: Oestridae: Oestrinae) are obligate parasites of vertebrates responsible for causing cavitary myiasis (oestrosis) in sheep and goats. The sheep nose bot fly is a cosmopolitan parasite, and adult flies can be found, especially in the Mediterranean areas, Europe, Africa, and some tropical areas with warm climates (Meleney et al., 1962; Horak, 1977; Dorchies et al., 2000; Scala et al., 2002; Shoorijeh et al., 2009, Gracia et al., 2010). Sneezing and nasal discharges are the most prominent clinical symptoms (Horak, 2005). Overall, the damaging effect on the nasal sinuses can cause other serious problems for the animal's health and consequently impair animal production (Ahaduzzaman, 2019) with economic losses. Infestation with larval stages of *O. ovis* has been described as a zoonotic disease that leads to ophthalmomyiasis and nasopharyngeal myiasis in humans (Brini et al., 2019; Ahmed et al., 2022).

In Brazil, studies on the epidemiology of *O. ovis* are scarce (Ribeiro et al., 1990; Ramos et al., 2006; Silva et al., 2012, 2013) and limited to the southern and southeastern regions of the country and restricted to the Pampa, Cerrado, and Atlantic Forest biomes. In the Midwestern region, the occurrence of oestrosis within the Amazon biome is limited to an outbreak in sheep (Schenkel et al., 2012) and a record of *O. ovis* parasitizing goats and sheep in the Cerrado of the Brazilian central plateau (Cansi et al., 2011). Therefore, the development of studies can be relevant from a veterinary perspective for designing an appropriate control strategy (Nilssen, 2006), considering the variation in infestation according to seasonality. Thus, the present study aimed to investigate the seasonal prevalence of *O. ovis* among slaughtered sheep in the Northern Region of the Amazon biome in the State of Mato Grosso, Brazil.

## Materials and Methods

**Figure 1:**
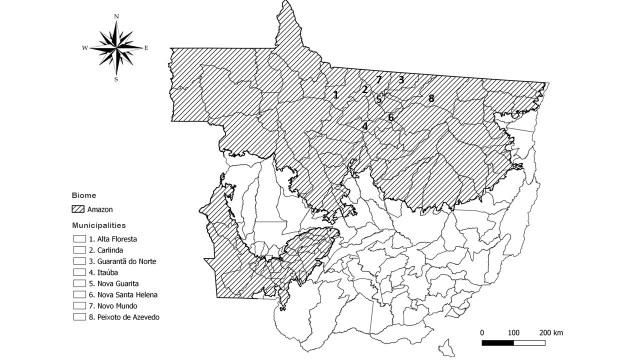
Municipalities from where sheep were sent to slaughter, between November 2011 and November 2013, within the Brazilian Amazonia, State of Mato Grosso, Brazil.

The heads of a total of 697 sheep that were sent to a slaughterhouse located in the municipality of Terra Nova do Norte (10°31′01″ S, 55°13′51″ W), between November 2011 and November 2013 were made available for the present study. The heads of the slaughtered animals were from eight municipalities throughout Northern of Mato Grosso, within the Amazon biome of the state of Mato Grosso, Brazil (Figure 1).

Sheep were slaughtered randomly regardless of sex, age, or location. All heads were removed, frozen, and sent to the Animal Pathology Laboratory of the Federal University of Mato Grosso in the municipality of Sinop, where they were defrosted and sagittally divided using a bandsaw. For each antimere, all larvae were collected, counted, and cataloged according to location. They were then stored in 70% alcohol, identified, and separated according to the larval stage using identification keys (Cepeda-Palacios et al., 1999; Colwell, 2006).

Prevalence, intensity of parasitism, and abundance were calculated according to Bush et al. (1997). Subsequently, prevalence, mean intensity, and mean abundance were compared with the monthly mean humidity and mean temperature (Agritempo, 2021). The Pearson coefficient was calculated, and linear regression analysis was performed with humidity and temperature as the independent variables, and prevalence, mean intensity, and mean abundance as the dependent variables. All analyses were performed using the R statistical package (R Development Core Team, 2021).

## Results

The analysis included a total of 697 sheep heads from eight municipalities. The number of sheep slaughtered per month varied during the 24 months (Table 1) owing to the demand for sheep meat during the present study period. Thus, the month with the highest number of heads analyzed was November 2013 (n=118) and the lowest was February 2012 (n=2). As the slaughter was dependent on demand, there were no sheep slaughtered in April 2013 and the month was excluded from the analysis (Table 1). The average number of animals slaughtered and analyzed was 29.04 heads/month, taking into account only the 24 months in which sheep were slaughtered.

**Table 1:** Occurrence of *Oestrus ovis* in sheep slaughtered between November 2011 and November 2013, within the Brazilian Amazonia, State of Mato Grosso, Brazil, with data of the average relative humidity and average temperature for the period.

**Year**	**Month**	**N**	**N_+_**	**Prevalence (%)**	**N° Larva**	**Mean Intensity**	**Mean Abundance**	**Mean Humidity (%)**	**Mean Temperature (°C)**
2011	Nov	70	16	22.9	51	3.19	0.73	78.8	25.2
	Dec	48	32	66.7	151	4.72	3.15	79.7	25.6
									
2012	Jan	4	2	50	30	15	7.5	80.9	24.9
	Feb	3	2	66.7	3	1.5	1	76.9	25.1
	Mar	21	13	61.9	94	7.23	4.48	81	25.1
	Apr	19	8	42.1	14	1.75	0.74	79.8	25.8
	May	10	7	70	25	3.57	2.5	76.3	24.6
	Jun	14	8	57.1	21	2.63	1.5	69.5	24.8
	Jul	30	16	53.3	188	11.75	6.27	57.5	24.8
	Aug	31	8	25.8	47	5.88	1.52	46.7	26.4
	Sep	10	5	50	84	16.8	8.4	56.9	28.4
	Oct	17	13	76.5	158	12.15	9.29	66.9	27.9
	Nov	24	18	75	297	16.5	12.38	75.4	26.5
	Dec	35	22	62.9	202	9.18	5.77	75.2	26.4
									
2013	Jan	24	12	50	77	6.42	3.21	82.4	25.4
	Feb	30	10	33.3	33	3.3	1.1	85.6	24.8
	Mar	62	24	38.7	175	7.29	2.82	87	25.6
	May	17	12	70,6	76	6,33	4,47	70	26
	Jun	18	0	0	0	0	0	66.2	26.1
	Jul	32	15	46.9	237	15.8	7.41	58	25.1
	Aug	19	13	68.4	121	9.31	6.37	50.1	25.9
	Sep	20	7	35	34	4.86	1.7	58.8	28
	Oct	21	6	28.6	11	1.83	0.52	68.2	27.5
	Nov	118	50	42.4	211	4.22	1.79	76.6	26.2

Overall, 45.77% (319/697) of the animals were infested with 2,412 recovered larvae, of which 96.89% (2,337/2,412) were identified at the species level as *O. ovis*. Among the collected larvae, 37.31% (872/2,337), 42.49% (993/2,337), and 20.2% (472/2,337) were in the first, second, and third *O. ovis* larval stages, respectively. Based on the location of the recovered larvae, 58.58% (1,413/2,412) were found in the common nasal meatuses, followed by 23.26% (516/2,412) in the frontal sinuses; 10.12% (244/2,412), 2.86% (69/2,412), and 2.20% (53/2,412) in the ventral, dorsal, and ethmoidal nasal meatuses, respectively; 1.95% (47/2,412) in the maxillary sinuses, and 1.04% (25/2,412) in the middle nasal meatuses. The minimum and maximum prevalence was observed in November 2011 (22.9%) and October 2012 (76.5%), respectively, with a mean of 12.76 positive heads/month (± 7.92 standard deviation).

**Figure 2:**
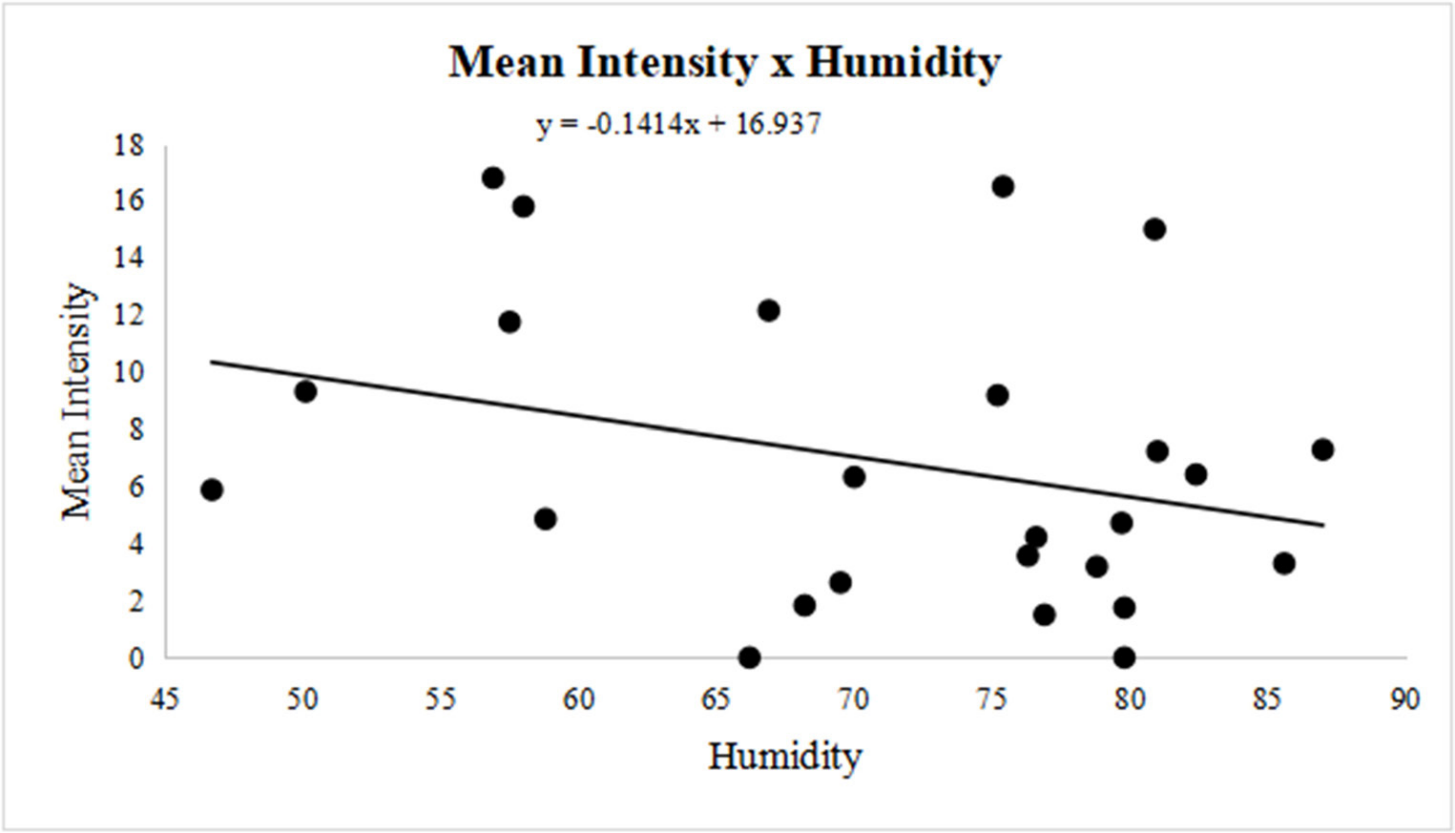
Variation in the mean humidity and mean intensity of infection (r = -0.2745) of *Oestrus ovis* in the surveyed part of the Eastern Amazon, Brazil, between November 2011 and November 2013.

**Figure 3:**
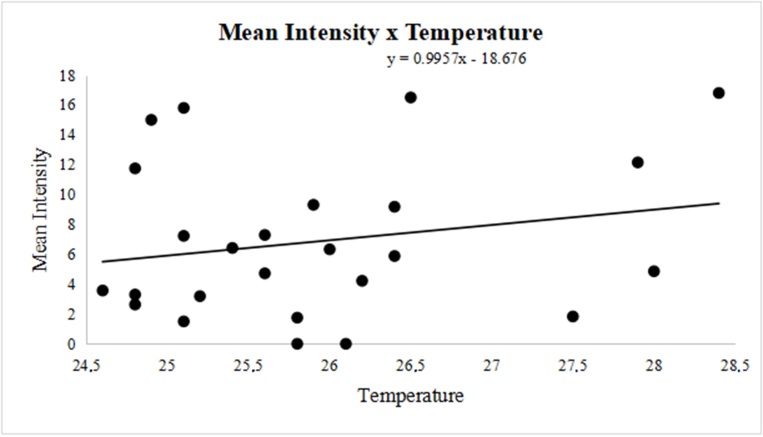
Variation in the mean temperature and mean intensity of infection (r = 0.2129) of *Oestrus ovis* in the surveyed part of the Eastern Amazon, Brazil, between November 2011 and November 2013.

The seasonal variation in prevalence ranged from 41% (spring - from September to December) to 56% (summer - from December to March). Still, there was no correlation between prevalence and season, mean humidity, or temperature, based on linear regression analysis and calculation of the Pearson’s coefficient. However, the mean intensity of parasitism was negatively correlated with mean humidity (r = -0.2745), demonstrating that higher humidity decreased mean intensity (Figure 2). Furthermore, we observed a positive correlation with mean temperature variation (r = 0.2129), indicating that higher temperatures increased the mean intensity (Figure 3).

**Figure 4:**
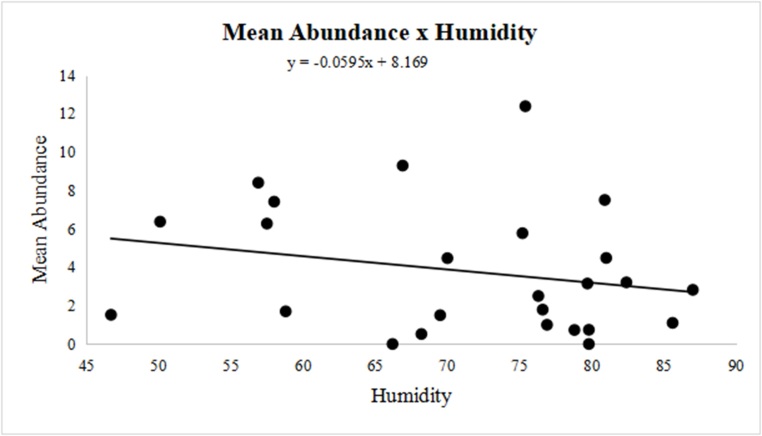
Variation in the mean humidity and mean larval abundance (r = -0.2033) of *Oestrus ovis* in the surveyed part of the Eastern Amazon, Brazil, between November 2011 and November 2013.

**Figure 5:**
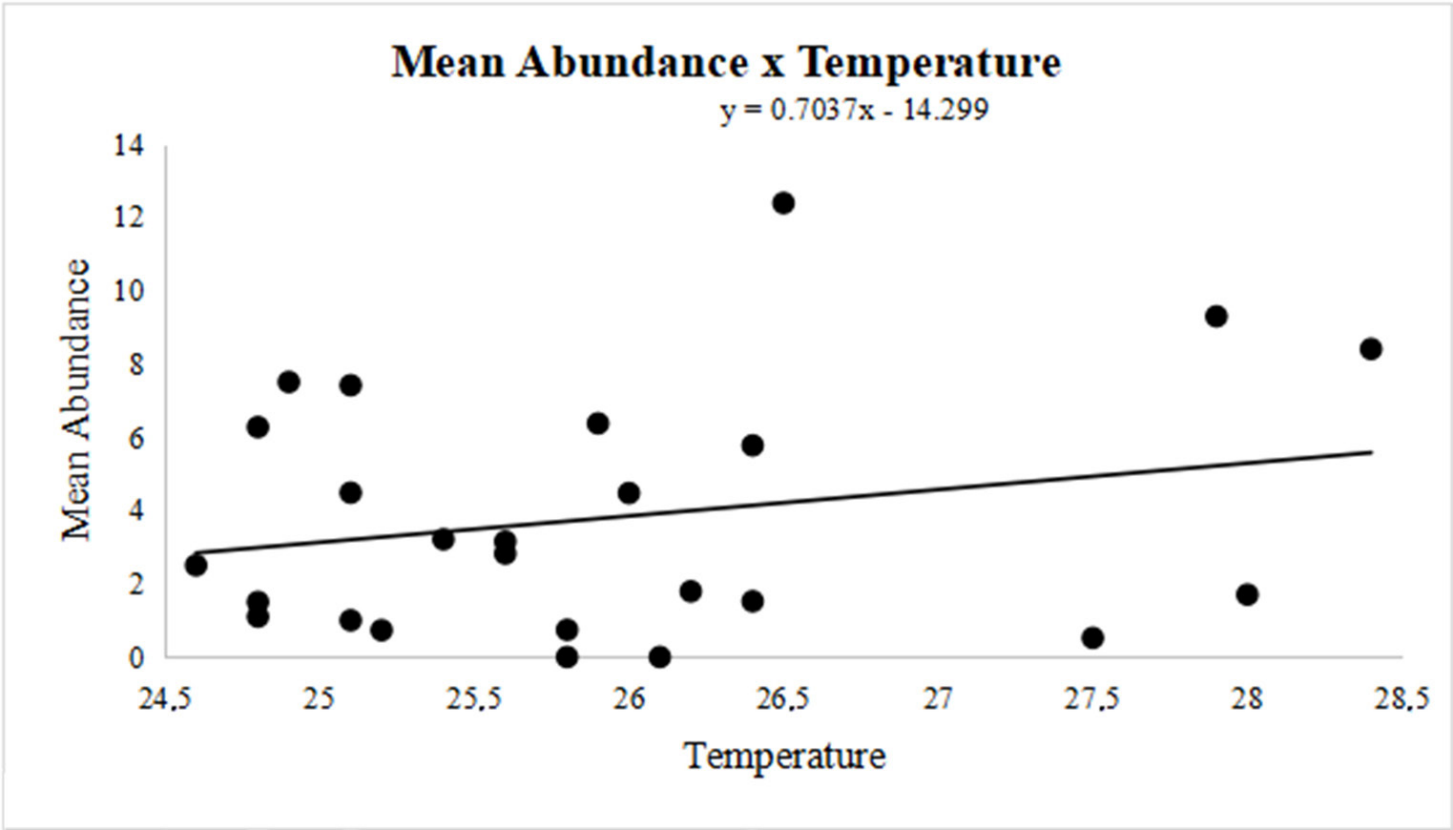
Variation in the mean temperature and mean larval abundance (r = 0.2328) of *Oestrus ovis* in the surveyed part of the Eastern Amazon, Brazil, between November 2011 and November 2013.

Similar to the observations related to the mean intensity of parasitism, the mean abundance of larvae was negatively correlated with the mean humidity (r = -0.2033), demonstrating that higher humidity decreases the mean abundance (Figure 4). Finally, a positive correlation with mean temperature variation (r = 0.2328) showed that higher temperatures increased mean abundance (Figure 5).

## Discussion

The prevalence (45.77%) of *O. ovis* infection in the present study differed from that in similar studies carried out in other countries. The prevalence observed in the present study was lower than those reported from Spain, where 71.1% of the sheep were infested with *O. ovis* larvae (Alcaide et al., 2003); or even in Sicily with a prevalence of 55.8% (Caracappa et al., 2000) and Greece with 54% of the animals infested (Papadopoulos et al., 2010). However, the prevalence was higher than those reported in Turkey where 364 sheep heads were surveyed and 22.52% were infested with *O. ovis* (Karatepe et al., 2014), and Libya with a prevalence of 42.33% (Negm-Eldin et al., 2015). Moreover, the values for the present study differed from those reported for areas within Brazil at 13.7% (Silva et al., 2013). Despite these individual differences, the prevalence found in the present study was within the reported global average (51.1%) (Ahaduzzaman, 2019). Several environmental factors can affect the prevalence and intensity of *O. ovis* infections in sheep and interannual variations are not rare (Tabouret et al., 2001) the results obtained for the infection rate could be due to these environmental conditions of the areas. It should be noted that the fly occurs year-round in regions where temperature and humidity remain constant, similar to what was found by Silva et al. (2012, 2013), who demonstrated a possible correlation with the maintenance of temperature and humidity at a certain level throughout the year, a characteristic observed in the southeastern Amazon region. Contrary to previous reports, regions with temperate climates have a lower prevalence than tropical regions (Tabouret et al., 2001).

*Oestrus ovis* can cause severe parasitosis in sheep and goats but can occasionally infect other species of animals (Horak, 2005). The host response to oestrosis and the larval burden is related to several factors, such as the susceptibility of host species, chronobiology of *O. ovis* in a particular geographical region, routine animal management practices (Sotiraki & Hall, 2012), temperature, and humidity. These larvae are obligatory parasites of the nasal cavity and sinuses (Ahaduzzaman, 2019). Newly deposited first-stage larvae actively migrate to the nasal passage and attach to the mucous membranes (Scala et al., 2002). This migration could be the reason for the differences found in each region during necropsies. Other studies conducted in slaughterhouses in different countries revealed infestation of *O. ovis* larvae from the nasal sinuses of sheep and goats (Benakhla et al., 2004; Attindehou et al., 2012). Larvae are most widely found in the nasal cavity (Yacob et al., 2004), as observed in the present study. In contrast, the distribution of the abundance of larval stages in our study was similar to that reported by Dorchies & Alzieu (1997), which is necessary for the development of oestrosis throughout the year.

Studies on the seasonality of oestrosis in the Amazon are scarce, however, when one takes into account that the use of prophylactic and curative medications does not eliminate all larvae (Bello et al., 2022), understanding the biology of larvae in each region becomes extremely important. Oestrosis is reported as a disease with increased occurrence in hot and dry regions (Gracia et al., 2019), so this explains the mean intensity of parasitism and the mean abundance of larvae observed in our study. The higher the temperature and lower the humidity, the greater the intensity and abundance observed (Figures 2 and 3). Our results indicate that parasitism by *O. ovis* in sheep occurs year-round, taking into account the occurrence of larvae (L1, L2, and L3) throughout the year.

In Brazil, a study in Ituiutaba in the Southeast region, a predominant Cerrado biome, highlighted the increased severity of the occurrence associated with high temperatures and low pluviometric rates (Magalhães et al., 2021), which is in agreement with another study conducted in America by Fonseca et al. (2018). Therefore, we believe that a more hostile environment interferes with the larviposition habits of females, as well as with the activity of the fly, larval development, and pupal development on the ground (Silva et al., 2012). Temperature and humidity are key to understanding the behavior of these flies because they are directly related to their activity (Cepeda-Palacios & Scholl, 2000).

The pathogenesis of *O. ovis* is correlated to the mechanical trauma and irritation from cuticular spines and oral hooks, in addition to enzymes and antigens excreted or secreted by the larvae that induce a hypersensitivity immune reaction (Gracia et al., 2019). *Oestrus ovis* induces massive recruitment and degranulation of mast cells, especially L2 infection in the septum to the ethmoid sinus (Yacob et al., 2004), and in this sense, neither infestations nor the formation of granulomas was identified in this anatomical region. However, we emphasize that the presence of *O. ovis* leads to breathing difficulties, decreases grazing activity and rumination time, and has negative nutritional effects including general malnutrition and low performance (Gracia et al., 2019).

Concerning control and prevention, several anthelmintics, such as ivermectin or closantel, are effective against sheep nasal bot flies (Bello et al., 2022). However, the widespread use of medications has been leading to resistance issues. In this sense, new biotechnologies, such as biological control (Weeks et al., 2018), or even infrastructure and waste management are viable alternatives. Although oestrosis primarily occurs in sheep and goats (Rao et al., 2018), it is the most common cause of ophthalmomyiasis in humans (Brini et al., 2019; Ahmed et al., 2022), with ophthalmic and nasopharyngeal reports in humans (Panadero-Fontán & Otranto, 2015) and dogs (Lucientes et al., 1997). Considering the scope of One Health (WHO, 2023), this feature of the constant maintenance of flies and larvae could potentially increase the risk of zoonosis and influence the occurrence of human infection (Ahaduzzaman, 2019) in these areas, and it is an alert for workers in the region.

## Conclusions

In conclusion, parasitism by *O. ovis* in sheep in the northern region of the state of Mato Grosso, occurs year-round, taking into account the occurrence of larvae (L1, L2, and L3) throughout the year, probably because of the area's environmental conditions. Thus, the lack of such studies in the Amazon region indicates that further studies are needed to improve the sanitary management of sheep and goats to reduce disease losses and to evaluate the influence of oestrosis on sheep productivity in these environmental conditions.
